# Convergent Evolution of Silk Webbing in Eriophyoid Mites (Eriophyoidea) and *Aceria*–*Cisaberoptus* Sympatry on Mango

**DOI:** 10.3390/insects17030259

**Published:** 2026-02-28

**Authors:** Philipp E. Chetverikov, Charnie Craemer, Alexey G. Desnitskiy, Nikita S. Kopylov, Andrey T. Kudrjavtzev, Viert D. Nguen, Anna E. Romanovich, Hoat X. Trinh, Andrey E. Vishnyakov, James Amrine

**Affiliations:** 1 Department of Invertebrate Zoology, St. Petersburg State University, Universitetskaya Nab. 7/9, 199034 St. Petersburg, Russia; adesnitskiy@mail.ru (A.G.D.); nikitakopylov146@gmail.com (N.S.K.); aromanovich@gmail.com (A.E.R.); vishnyakov@hotmail.com (A.E.V.); 2 Department of Parasitology, Zoological Institute of Russian Academy of Sciences, Universitetskaya Nab., 1, 199034 St. Petersburg, Russia; andrey.kudriavtsev@zin.ru; 3 Manaaki Whenua—Landcare Research, 231 Morrin Road, Auckland 1072, New Zealand; charniecc@gmail.com; 4 Department of Entomology, Plant Protection Research Institute, Dong Ngac, Hanoi 11909, Vietnam; viet.nguyenducvfc@gmail.com (V.D.N.); trinhxuanhoatppri@gmail.com (H.X.T.); 5 Division of Plant & Soil Sciences, West Virginia University, P.O. Box 6108, Morgantown, WV 26506, USA; james.amrine@mail.wvu.edu

**Keywords:** *Mangifera indica*, *Lepisanthes*, silk producing organs, arthropod structure, phylogeny

## Abstract

Several lineages within the Eriophyoidea, an ancient group of phytoparasitic acariform mites, produce large protective silk webs on plant surfaces. This study examined the diversity and phylogeny of web-spinning eriophyoids associated with the leaves of *Mangifera indica* (mango) in Asia, Africa, and North America and *Lepisanthes rubiginosa* (mertajam) in Vietnam. We report, for the first time, silk-producing structures in *Aceria aegyptindicae* and *Aculops knorri*, as well as the presence of tibial seta *l’* I and a lobular unpaired prosomal gland in *Cisaberoptus kenyae*. Molecular and morphological data indicate female dimorphism in *A. knorri* and reveal cryptic diversity within *Cisaberoptus* and mango-associated *Aceria*. The web-spinning species *C. kenyae* and *Ac. aegyptindicae* form a strongly supported clade and frequently coexist in syntopy on mango leaves, likely co-producing silk web. Phylogenetic analyses of COI and 28S genes place this clade as sister to Cecidophyinae, while grouping *Aberoptus* and *A. knorri* with Anthocoptini and Aceriini. These results demonstrate convergence, cryptic speciation, and unexpected mutualism in web-spinning eriophyoids, providing a framework for future research on the evolution of silk producing organs in this economically important mite group.

## Simple Summary

Eriophyoid mites are an ancient lineage of highly specialized plant parasites. Despite their minute size, certain species produce large protective silk nests. Using an integrative approach combining field observations, detailed morphology, and molecular data, we investigated web-spinning mites associated with the leaves of *Mangifera indica* (mango) in Asia, Africa, and North America and *Lepisanthes rubiginosa* (mertajam) in Vietnam. Our results demonstrate that silk-web production evolved convergently in phylogenetically distant lineages. We also show that on mango, the species *Cisaberoptus kenyae* and *Aceria aegyptindicae* frequently co-occur and likely cooperate in spinning shared webs. Finally, we provide evidence for a phylogenetic relationship linking the mango-associated web-spinners to the mite subfamily Cecidophyinae.

## 1. Introduction

The superfamily Eriophyoidea is an exceptionally ancient lineage of highly host-specific and morphologically simplified phytoparasitic acariform mites [1,2]. Phylogenetic reconstructions integrating fossil evidence and plant evolution suggest that, following their Devonian origin ~376 (314–441) Ma, eriophyoid mites underwent a transitory phase associated with the roots of early arborescent plants, co-evolved with now-extinct plant lineages, and later colonized gymnosperms and angiosperms [3,4,5,6]. The estimated divergence time of the crown group of Eriophyoidea, ~305 (260–364) Ma, closely aligns with the emergence of early conifers [6] and supports the hypothesis that gymnosperms were the primary host group for the radiation of extant eriophyoid lineages [3,7].

Current evidence indicates that the morphological evolution of Eriophyoidea is characterized by extensive convergence and homoplasy [2,8,9,10]. Key patterns include widespread morphological modifications like setal loss, dorsal opisthosomal transformations including the formation of ridges, furrows and pseudotagmata, structural variety of tarsal empodia, and leg segment fusion [1,11,12]. This is contrasted by divergence at higher taxonomic levels, defined by specific chaetotaxy of the prodorsal shield and topography of external and internal genitalia [2,13,14]. Speciation is frequently cryptic and driven by host shifts, which typically occur within a single plant genus or family, leading to complexes of subtly differentiated species sharing a similar design of prodorsal shield [9,15,16,17]. As a result, species delimitation usually requires the integration of detailed morphometric data with barcode gene comparisons [18,19,20,21,22,23].

The ability to produce large film-like silk webbing on plant surfaces (web-spinning) has long been considered an exotic adaptation that evolved in a few eriophyid taxa [24]. A series of recent publications has documented the presence of putative silk-producing structures in multiple unrelated lineages of Eriophyoidea, suggesting that this capability is more widespread [25,26,27,28,29]. Although the anatomy of gall mites is relatively well-studied [30], the detailed structure of their silk-producing organs has been examined in only a single species, *Aberoptus schotiae* Chetverikov et al. 2023 [27]. Current hypotheses for silk evolution and function in eriophyoid mites suggest that: (1) the silk-secreting system evolved from a symplesiomorphic complex of internal organs, including the anal glands, hindgut, and associated cuticular sacs; (2) this system is well-developed in taxa that produce large webs but reduced yet still functional in other species; (3) the web nests serve to regulate the micro-environment, protect mites from predators and pathogenic fungi, prevent dislodgement by rain, maximize occupation of the leaf surface, and isolate food resources from competitors; and (4) beyond web formation, the silk secretions of the anal glands may also enable attachment to plant surfaces, aid in ballooning during aerial dispersal, mark selected sites on the host, and facilitate intraspecific signaling to help conspecifics locate each other.

To date, ten species from four genera in the subfamilies Aberoptinae (*Aberoptus* Keifer, 1951 and *Cisaberoptus* Keifer, 1966), Phyllocoptinae (Anthocoptini: *Aculops* Keifer, 1966) and Eriophyinae (Aceriini: *Aceria* Keifer 1944) of the family Eriophyidae have been shown to produce large webs [24,27]. Nine of them are associated with arboreal host plant genera of the families Fabaceae, Ochnaceae, Anacardiaceae, and Sapindaceae (all within the eudicot superclade “rosids”), exhibiting pantropical and subtropical distributions and growing naturally across the Americas, Africa, Asia, and Australasia within frost-free, warm climates [27]. One species, *Aceria gersoni* Manson 1984, was described from the North Island of New Zealand living under webbing on the underside of the pinnules on the Rough tree fern, *Dicksonia squarrosa* (G. Forst.) Sw. (Polypodiopsida: Cyatheales: Dicksoniaceae) [31].

The genus *Aberoptus* comprises seven described species associated with plants in the Fabaceae (5 spp.), Ochnaceae (1 sp.), and Anacardiaceae (1 sp.), and reported from Brazil, South Africa, Taiwan, and Samoa. Female dimorphism has been confirmed in at least three species of *Aberoptus* (*Ab. cerostructor* Flechtmann 2001, *Ab. inusitatus* (Britto et al. 2008), and *Ab. schotiae* Chetverikov et al. 2023) through experimental studies [25,32] and genetic analysis [27]. In these species, the web-spinning form is specialized morphologically, featuring greatly modified swollen legs with heteromorphic empodia I and II, a spatulate structure on tarsus I, and two shortened terminal leg segments. The alternative female form resembles a typical *Aceria*, though it shares some general characteristics with the web-spinning form, such as swollen legs and a similar body shape.

The monotypic genus *Cisaberoptus* is well-known for its web-spinning pest species, *C. kenyae* Keifer, 1966, infesting mango (Anacardiaceae: *Mangifera indica* L.). It is present in most mango-cultivating regions worldwide, including southern Europe [33,34,35]. This species exhibits distinctive morphology, including a spatulate terminal palp segment forming a sucker-like structure; swollen legs I and II with large, pad-like empodia bearing a large number of rays (15–18); partially fused genua and femora; shortened tarsi and tibiae; and a putatively absent tibial seta *l*′ I. However, the functional correlation of this morphology with web-spinning behavior remains unknown. Although no specialized silk-producing structure has been described in *Cisaberoptus*, three hypotheses on the mechanism of silk production have been proposed: (1) regurgitation of intestinal content, (2) secretion from the salivary glands, or (3) production by anal glands and deposition via the hindgut [24,27,35,36]. Studies from Africa (Sudan, Egypt), Asia (Thailand), and South America (Brazil) have consistently reported *Aceria*-like specimens within the web nests of *C. kenyae* [34]. Researchers from Brazil interpreted these as a protogyne form of *C. kenyae* [37], whereas those working with Egyptian material described them as a new inquiline species, *Aceria aegyptindicae* Elhalawany et al. 2021 [34].

*Aculops knorri* Keifer, 1976 was described from Thailand, where it was found beneath a web coating on the leaves of *Lepisanthes rubiginosa* (Blume) Leenh. (Sapindaceae), mertajam, in tropical Asia and northern Australia [38]. Although serological tests confirmed the webbing as true proteinaceous silk [39], detailed anatomical data explaining how *A. knorri* produces silk are completely lacking. Apart from slightly swollen legs (primarily due to thickened femora), no specialized morphology correlated with its web-spinning ability has been reported. Taxonomically, *A. knorri* fits well within the tribe Anthocoptini (Eriophyidae: Phyllocoptinae). The species’ author noted that in the absence of specialized morphology it “… can only be assigned to the ‘waste-basket’ genus *Aculops*” [38]. This is a large anthocoptine genus comprising ~200 described species, which appears polyphyletic in molecular studies [9,40]. Remarkably, *Aceria* is another ‘waste-basket’ genus in Eriophyoidea, containing over 1000 species. Earlier authors proposed synonymies among *Aceria*, *Cisaberoptus*, and *Aberoptus* [11,32,36,37]; however, most recent studies consider them three valid genera [26,27,32,35].

No molecular data on web-spinning species of *Aceria* or *Aculops* are currently available. A single phylogenetic study focused on *Aberoptus* and *Cisaberoptus* recovered three 28S sequences of these genera in unrelated clades within Eriophyidae [27].

In this study, we (1) describe our field observations of populations of web-spinning mites associated with *L. rubiginosa* and *M. indica*, (2) investigate their genetic diversity using COI and 28S sequences, (3) report novel morphological characters observed in these mites, (4) test the conspecificity of *C. kenyae* and *Ac. aegyptindicae*, and (5) assess the monophyly of the ecological group of web-spinning eriophyid taxa based on an expanded dataset.

## 2. Materials and Methods

**Figure 1:**
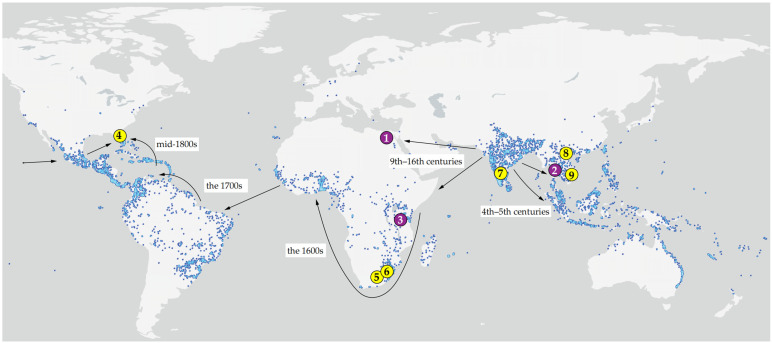
Type localities (1–3, purple) and source localities of the studied material (4–9, yellow) of web-spinning eriophyoid mites from *Mangifera indica* (1, 3–8) and *Lepisanthes rubiginosa* (2, 9), mapped onto the global distribution of mango based on GBIF [42] Occurrence Download. Available online: https://www.gbif.org/species/3190638 (accessed on 10 January 2026). Arrows indicate inferred routes and chronology of the human-mediated migration of mango following its domestication in India c. 4000 BP (adapted from [43]). 1—Type locality of *Aceria aegyptindicae*; 2—Type locality of *Aculops knorri;* 3—Type locality of *Cisaberoptus kenyae*; 4, 5, 6, 7, 8—Collecting sites of mango mites in Florida (4), South Africa (5, 6), India (7), and Vietnam (8); 9—collecting site of *Aculops knorri* in Vietnam. Note: Specimens from locality 7 (India) were not available for examination; the sequence MW491351 was included in the molecular analysis. Additional information is given in the Table 1.

**Collecting and slide-mounting.** Web-spinning eriophyoid mites were collected from two eudicot tree species from Vietnam, South Africa, and USA and used for morphological investigation and genotyping (Table 1, Figure 1). The web-coated leaves of *M. indica* (Anacardiaceae) and *L. rubiginosa* (Sapindaceae) were examined under a stereo microscope and the mites were collected from under the web using a minuten pin. Some mites were slide-mounted in Hoyer’s medium [41] and cleared on a heating block at 95 °C for 30–40 s (for obtaining temporary slides with partially cleared mites) or for 4–5 h (for obtaining permanent slides of fully cleared mites). Some mites were mounted alive in glycerin for investigating their anatomy in vivo. The rest of the mites were stored in Eppendorf tubes filled with 96% ethanol in a freezer (−25 °C) or kept alive on leaves in a refrigerator (+4 °C) for further examination.

**Mite populations used for microscopy observations.** Two populations (1 and 2, see below) of the web-spinning mites from Vietnam were used for stereo microscopy, phase contrast (PC), and differential interference contrast (DIC) light microscopy (LM), while additional populations from Tam Dao (Vietnam), Mtunzini (South Africa), and Florida (USA) (Table 1) were examined only under PCLM and were used for morphological comparison and molecular phylogenetic analyses.

Population 1: *A. knorri* under a web coating on leaves of *L. rubiginosa* collected in early March 2024 in a small forest between road QL29 and the coast of Vung Ro Bay, approximately 80 km north of Nha Trang City in Hoa Xuan Commune, Dak Lak Province, 12°52′43.8″ N 109°25′20.7″ E.

Population 2: Sympatric *C. kenyae* and *Aceria* spp. under a web coating on leaves of approximately 40-year-old *M. indica* trees growing near the entrance of the Plant Protection Research Institute collected in mid-February 2023 in Hanoi, 21°04′18.8″ N, 105°46′30.2″ E.

**Morphological measurements and comparison.** The external morphology of the slide-mounted specimens was examined using conventional light microscopy (PC LM and DIC LM) with a Leica DM2500, and via confocal laser scanning microscopy (CLSM) using previously described methodologies and equipment [18,44]. For comparative analysis, morphometric data for *A. knorri*, *C. kenyae*, and *Aceria* spp. from mango were compiled from the literature [34,37,38,45] and supplemented with new measurements from populations 1 and 2 (described above). The terminology for eriophyoid morphology and the classification scheme for Eriophyoidea adhere to [1,11].

**DNA extraction, PCR, and sequencing.** For DNA extraction, from one to eight mite specimens were crushed separately with a fine pin in a 2 μL drop of sterile water on a cavity well microscope slide. Each drop was pipetted into a thin-walled PCR tube with 25 μL of 6% solution of Chelex^®^ 100 Resin Bio Rad before being heated three times (5 min at 95 °C) in a thermostat with intermediate short vortexing. The solution above the Chelex^®^ granules was used as the DNA template for PCR to amplify the fragments of the nuclear *28S* rDNA and mitochondrial *COI* genes (Table 1). For the PCR and sequencing, we applied reported protocols and primers [29]. Sequences were obtained using BigDye Terminator v.3.1 chemistry (Applied Biosystems, Foster City, CA, USA) and a 3500xl Genetic Analyzer (Applied Biosystems). Trace files were checked and edited using GeneStudioTM Professional 2.2.0.0 (https://genestudio-pro.software.informer.com (accessed on 3 April 2023)). Additionally, we obtained a new COI sequence (PX794729) of *Ab. schotiae* using previously extracted DNA of the mites from the type population [27]. In order to test the genetic identity of the “narrower” and “wider” specimens from population 1 of *A. knorri* from *L. rubiginosa* (see Section 3.1.1 and Section 3.2.1), we obtained four DNA isolates representing only “wider specimens” (isolates d633 and d1057), only “narrower specimens” (d1059) and their mixture (d712, Table 1).

**Analysis of COI and 28S sequence diversity.** Prior to this study, the only available sequences for *C. kenyae* were a 28S sequence (KT070272; 1014 bp) from South Africa [14] and a single unpublished COI sequence (MW491351; 634 bp) from specimens collected by P. Dyamanagouda on 10 October 2019 in Coimbatore, India (Tamil Nadu Agricultural University, Paddy Breeding Station; 11°00′ N, 76°91′ E), from under a web coating on leaves of *M. indica*. Both reference sequences were aligned with our newly generated sequences (Table 1), trimmed to equal length, and analyzed using the Kimura 2-parameter (K2P) model in MEGA7 [46] to estimate evolutionary divergence.

**Molecular phylogenetic analyses.** Approximately 3400 COI sequences of Eriophyoidea were available in GenBank as of 15 January 2026. Most are short fragments of ~650 bp obtained with classical Folmer’s primers [47] or their variants, alongside a smaller series of ~1200 bp sequences amplified with primers developed by Klimov et al. [48] and complete COI sequences derived from recent mitogenomic studies [40,49]. Phylogenetic reconstructions using all available COI sequences from GenBank did not converge and yielded biologically inconsistent topologies, likely due to the high proportion of short fragments with insufficient phylogenetic signal.

To overcome this issue, we performed a BLAST+2.17.0 search with sequence PX794735 (*C. kenyae*, 1196 bp) against Eriophyoidea in GenBank, downloaded all sequences with query coverage 90–100%, removed erroneous sequences [49] and duplicates, excluded sequences from Phytoptidae and Nalepellidae (except for the outgroups specified below), and supplemented the selection with new sequences (Table 1). The resulting curated sequences were codon-aligned using MUSCLE in MEGA 7 [46] and translated to amino acids to screen for stop codons. The final COI dataset included 193 sequences and 456 amino acid positions.

To construct the 28S dataset, we conducted a BLAST search with sequence PX789905 of *C. kenyae* against GenBank entries for Eriophyidae, downloaded all sequences with 55–100% query coverage, and merged them with our novel sequences (Table 1) and outgroups. The 28S sequences were aligned using the E-INS-i algorithm in MAFFT, accessed via the web server [50], with default parameters. The final 28S dataset included 112 sequences and 1993 nucleotide positions.

Outgroup sequences (COI, 28S) for seven genera of Phytoptidae s. str. (*Fragariocoptes, Oziella, Novophytoptus*) and Nalepellidae (*Boczekella*, *Nalepella*, *Setoptus*, *Trisetacus*) were sourced from previous studies [10,40,48].

Maximum likelihood (ML) analyses were performed using IQ-TREE 2.2.2.6 [51]. The best-fit substitution models mtART + R6 (COI, amino acids) and GTR + F + R5 (28S) were selected via ModelFinder [52] within IQ-TREE based on the Bayesian Information Criterion. Branch support was assessed using the Ultrafast bootstrap approximation (UFBoot) with 10,000 replicates, 1000 maximum iterations, and a minimum correlation coefficient of 0.99. The resulting ML trees were annotated with values of UFBoot and approximate likelihood ratio test (aLRT) calculated in IQ-TREE.

## 3. Results

### 3.1. Stereomicroscopy Observations of Web-Spinning Mite Populations on the Host Plant Organs

#### 3.1.1. *Aculops knorri* (Population 1)

**Figure 2:**
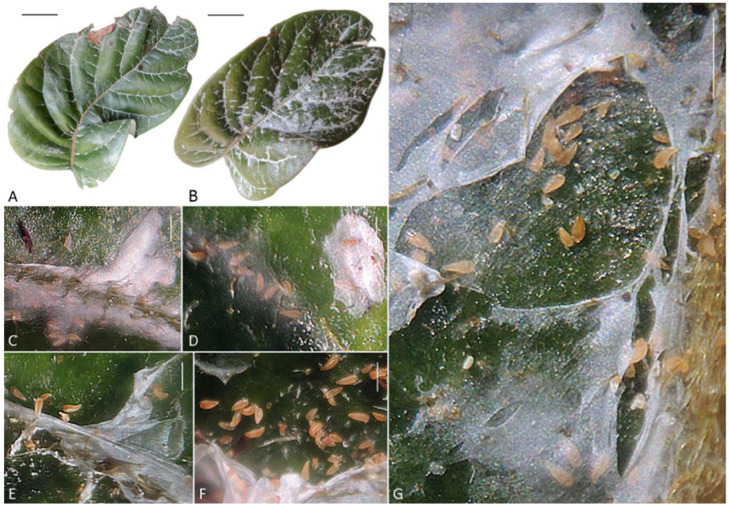
Web-nests of *Aculops knorri* on the upper surface of leaves of *Lepisanthes rubiginosa* (Central Vietnam). (**A**,**B**) Young (**A**) and old (**B**) leaf with web-coating; (**C**,**D**) mite clusters under young, semi-transparent webbing along the midrib (**C**) and secondary veins (**D**); (**E**–**G**) mite colonies under mature, dense white webbing between the veins (webbing partially removed to expose mites). Scale bar: (**A**,**B**)—1 cm; (**C**–**G**)—250 µm.

Most leaves on the sampled twigs of *L. rubiginosa* were covered with white webbing produced by *A. knorri.* On younger, bright green leaves located near the terminal buds, the webbing was less developed and restricted to the veins (Figure 2A). In contrast, on older leaves, the webbing was more extensive, covering larger areas, including the interveinal spaces (Figure 2B). The leaf epidermis beneath the webbing appeared similar to unwebbed areas and showed no evident signs of damage.

Small groups of approximately 5–10 mites were observed under a very thin, nearly transparent young web along the midrib and secondary veins on all infested leaves (Figure 2C,D). Within these groups, mites were predominantly oriented with their anal region toward the periphery of the webbing site, likely excreting silk through the spinnerets.

Among the females of *A. knorri* observed under a stereomicroscope, some individuals appeared slightly narrower and more elongated (“narrow” specimens) while others were slightly wider and shorter (“wider” specimens), indicating possible morphological femaledimorphism.

Mite density was higher on older leaves, where they formed large colonies containing brightly orange adults, light orange or whitish immatures, and eggs beneath notably denser and less transparent white webbing compared to younger leaves (Figure 2E–G). Approximately 2000 mites were washed with 96% ethanol from the single leaf shown in Figure 2B.

#### 3.1.2. *Cisaberoptus kenyae* and *Aceria aegyptindicae* (Population 2)

**Figure 3:**
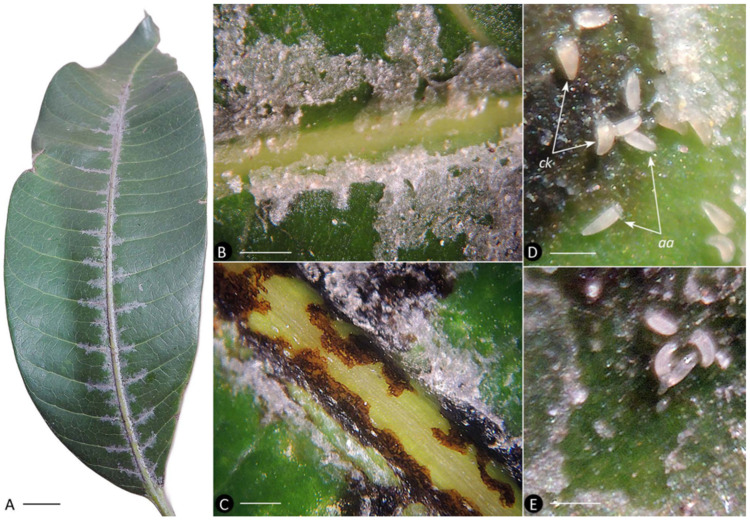
Webbing of eriophyoid mites on the upper surface of mango leaves. (**A**) Young leaf showing early mite webbing on the petiole, along the midrib and base of the secondary veins. (**B**) Semitransparent young webbing along the secondary vein of a young leaf. (**C**) Necrotic spots under webbing along the midrib of an older leaf. (**D**) Mixed colony of *Cisaberoptus kenyae* (*ck*) and *Aceria aegyptindicae* (*aa*). (**E**) Three adults and one nymph of *Ac. aegyptindicae* under young webbing. *Note*: webbing was partially removed (**C**–**E**) to expose necrotic spots and mites. Scale bar: (**A**) 1 cm; (**B**,**C**) 1 mm; (**D**,**E**) 200 µm.

The distribution of webbing observed on the mango leaves (Figure 3A) matched the pattern reported in the literature [34,35,36]. At an early stage, webbing was confined to areas near, but not directly covering, the veins. At a later stage, the webbing extended to cover the veins and interveinal spaces.

Irregular black spots, presumably of necrotic epidermis, were observed beneath the webbing. On younger leaves with lower mite density and early-stage infestation, these spots were confined to the epidermis adjacent to the veins (Figure 3B). On older leaves with heavier, more established infestations, the spots also occurred on the veins (Figure 3C).

Mixed colonies of two mite species, *C. kenyae* and *Ac. aegyptindicae*, were observed under the webbing on most investigated leaves. Adults of *C. kenyae* were light orange and slightly more triangular in shape than the adults of *Ac. aegyptindicae*, which were pale whitish and more elongated (Figure 3D,E).

*Remarks.* Beyond the mixed population 2 from Northern Vietnam, we repeatedly observed *C. kenyae* and *Ac. aegyptindicae* coexisting under the same webbing on mango leaves in two South African localities (Table 1). This co-occurrence aligns with previous reports of mixed colonies of *C. kenyae* and *Ac. aegyptindicae* from Sudan, Egypt, Thailand, and Brazil [34]. Overall, the current evidence indicates that *C. kenyae* and *Ac. aegyptindicae* frequently coexist under shared webbing, and such mixed colonies are widely distributed across mango-growing regions worldwide.

### 3.2. Microscopy Observations of Slide-Mounted Web-Spinning Mites

#### 3.2.1. Morphological Remarks on *Aculops knorri* (Population 1)

**Figure 4:**
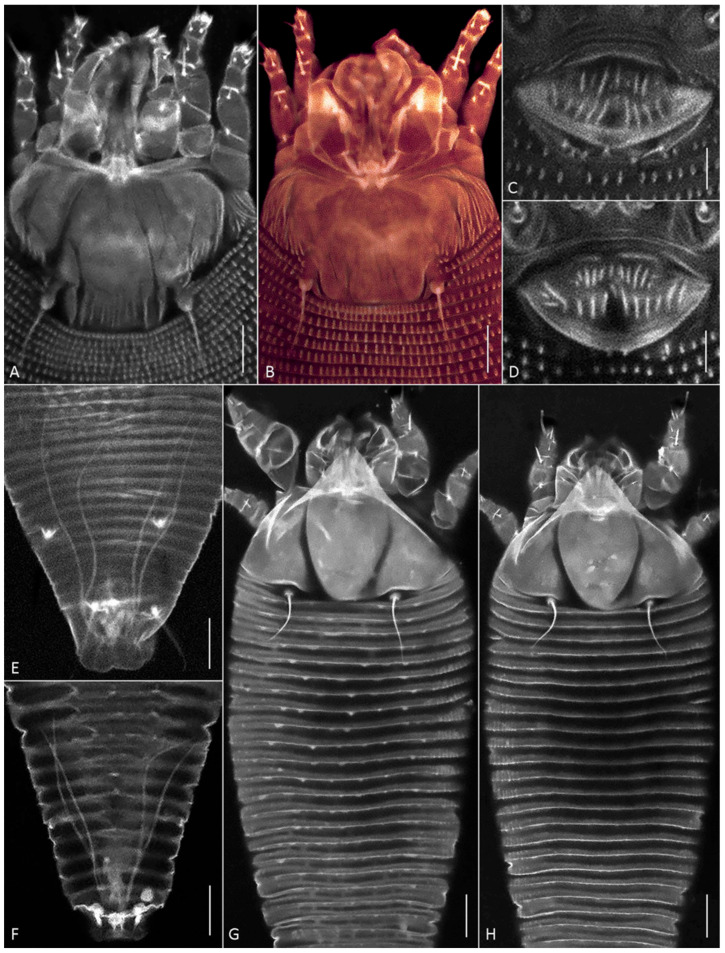
CLSM images of females of *Aceria aegyptindicae* (**A**–**E**) and *Aculops knorri* (**F**–**H**). (**A**–**D**) Prodorsal shields (**A**,**B**) and external genitalia (**C**,**D**) in *Ac. aegyptindicae*; (**E**,**F**) cuticular ducts of silk-producing anal glands; (**G**,**H**) dorsal view of prosoma and anterior part of opisthosoma in “wider” (**G**), and “narrower” (**H**) females of *A. knorri*. Note small subtriangular microtubercles on the dorsal opisthosomal annuli in Figure 4G. Scale bar: (**A**,**B**,**G**,**H**) 10 µm; (**C**–**F**) 5 µm.

While closely resembling the type Thai population [38], the studied Vietnamese specimens of *A. knorri* exhibited clear differences in prodorsal shield ornamentation and several morphometrics (Table S1). Contrary to the smooth prodorsal shield reported for the Thai specimens [38], the mites from Vietnam possess a prodorsal shield with two distinct, complete admedian lines, which delimit a central suboval area preceded by a large, broad-based, subtriangular frontal lobe (Figure 4G,H).

Within the Vietnamese population, females provisionally identified under a stereo microscope as “narrower” and “wider” forms (see Section 3.1.1.) differed in the following characteristics: the body width-to-length ratio, the lengths of setae *2a* and *l″ I*, and the ornamentation of the dorsal opisthosomal annuli (Table S1). The wider specimens possessed subtriangular microtubercles that were more pronounced on the anterior half of the opisthosoma. In contrast, all dorsal opisthosomal annuli were smooth in the narrower specimens (Figure 4G,H). These observations suggest the presence of two distinct morphotypes within the Vietnamese population of *A. knorri*.

#### 3.2.2. Morphological Remarks on *Cisaberoptus kenyae* (Population 2)

All investigated specimens of *C. kenyae* from our material possess a tibial seta *l′* I 3–4 µm long, a feature not reported by previous authors (Figure S1).

Compared to specimens of *C. kenyae* from the type population in Kenya [45], our specimens from Vietnam were slightly wider, had longer setae *sc*, and fewer empodial rays on leg II (Table S2).

**Figure 5:**
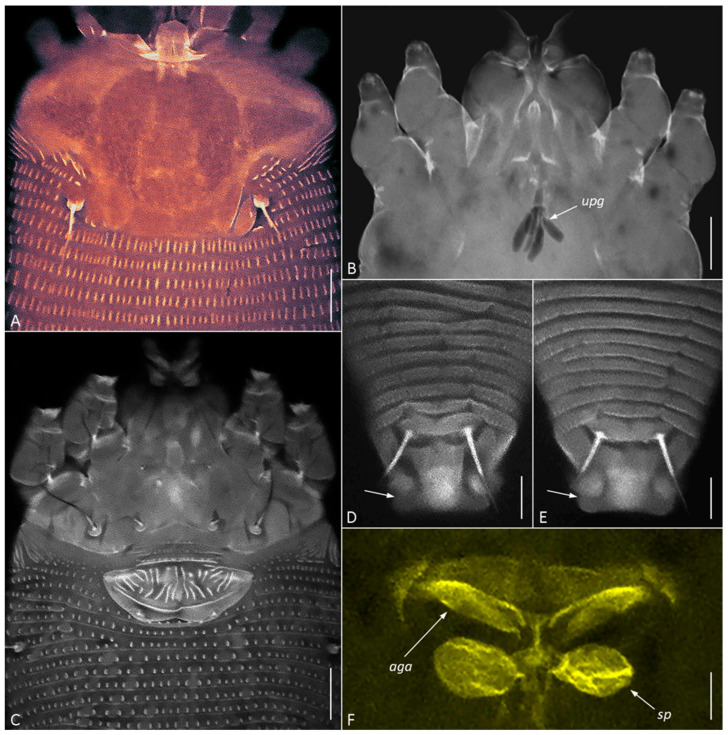
CLSM images of females of *Cisaberoptus kenyae* from Northern Vietnam (population 2). (**A**) Prodorsal shield with conspicuous frontal lobe. (**B**) Outlines of lobular unpaired prosomal gland (*upg*) outlined by auto-fluorescing partially dissolved internal tissues, (**C**) coxigenital area, (**D**,**E**) dorsal view of telosoma and caudal lobe with lateral folding (arrows), (**F**) internal genitalia including ovoid spermathecae (*sp*) and plate-like anterior genital apodeme (*aga*). Scale bar: (**A**–**C**) 10 µm; (**D**–**F**) 5 µm.

Specimens from Egypt [34] were morphometrically nearly identical to those from Kenya [45]. Notably, mites from both African populations (Kenya and Egypt) had a significantly narrower prodorsal shield than those in our material (Table S2). This difference, though consistent with geographical variation, is more likely caused by an artifact of measurement methodology or error since the recorded narrower prodorsal shield conflicts with the illustrated body proportions [34] (plate 1 DA); [45] (Figure 5D,F).

A morphometric investigation of an additional population of *C. kenyae* from Florida showed these mites to be slightly smaller, with shorter setae *sc* and *h2*, while matching the other morphometrics of specimens from Hanoi (Table S2).

CLSM of *C. kenyae* from Vietnam revealed a small subrectangular frontal lobe of the prodorsal shield 4–5 µm long (Figure 5A) and outlines of a large, unpaired gland associated with the motivator (Figure 5B, *upg*), a structure rarely observed under conventional light microscopy. A tiny cuticular lateral folding of the caudal lobe (Figure 5D,E, arrow) was registered in all specimens.

CLSM of the female genitalia of *C. kenyae* (Figure 5C,F) showed their general resemblance to those in members of the subfamily Cecidophyinae, which are characterized by external genitalia appressed to coxae II, a genital coverflap ornamented with short ridges organized in two transverse rows, and an anterior genital apodeme situated in a plane orthogonal to the main body axis and anterior to small ovoid spermathecae [11,53,54,55].

#### 3.2.3. Morphological Remarks on *Aceria aegyptindicae* (Population 2)

The Vietnamese specimens closely resembled those from the type population of *Ac. aegyptindicae* from Egypt and the specimens from Brazil, which were originally misidentified as protogynes of *C. kenyae* [34,37]. Morphometric differences among these three populations were minimal and largely overlapping (Table S3). Distinctive characters included: shorter setae (*ft″* I, *ft″* II, *l″* I, *l″* II, *1a*, *2a*, *d*, *e*) in Egyptian specimens; longer setae *c2* and *h2* in Vietnamese specimens; a greater number of ventral annuli and a larger distance between setae *sc* in Brazilian specimens; and a genital coverflap that was longest and narrowest in Egyptian specimens (Table S3).

The specimens from Vietnam showed variation in the ornamentation of the prodorsal shield and genital coverflap, delineating two putative forms. One form (identified as *Ac. aegyptindicae*) featured a prodorsal shield with distinct, numerous short lines laterally, a nearly smooth central area, and a genital coverflap with a greater number of basal ridges (Figure 4B,D). The other form (identified as *Aceria* cf. *aegyptindicae*) exhibited a shield with short subparallel lines medio-posteriorly and faint, longer lines resembling admedian or submedian lines, alongside a coverflap with notably fewer basal scores (Figure 4A,C).

#### 3.2.4. Silk-Producing Apparatus (SPA) of *Aceria aegyptindicae* and *Aculops knorri*

**Figure 6:**
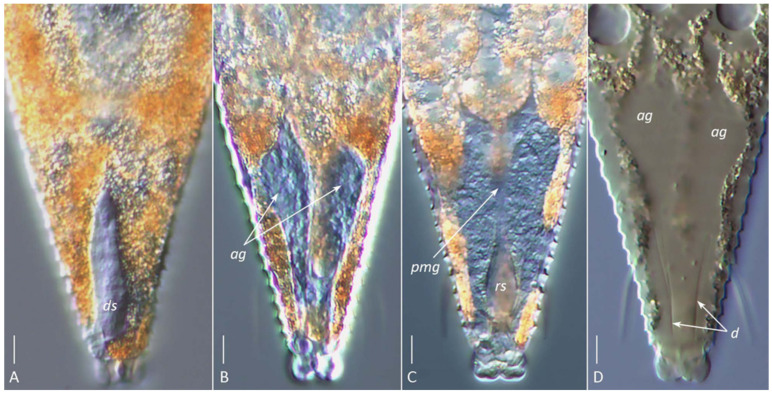
DIC images of the silk-producing apparatus in live ((**A**–**C**), glycerin mounts) and partially cleared ((**D**), Hoyer’s medium) females of *Aculops knorri*. (**A**) Dorsal sac (*ds*); (**B**) paired hypertrophied anal glands (*ag*); (**C**) rectal sac (*rs*, corresponding to the anterior part of the rectum) and putative posterior midgut (*pmg*); (**D**) outlines of partially dissolved anal glands (*ag*) and their cuticular ducts (*d*). *Note*: Images (**A**–**C**) are from different specimens, highlighting the dorsal (**A**), medial (**B**), and ventral (**C**) structures of the silk-producing complex. Scale bar: 5 µm.

**Figure 7:**
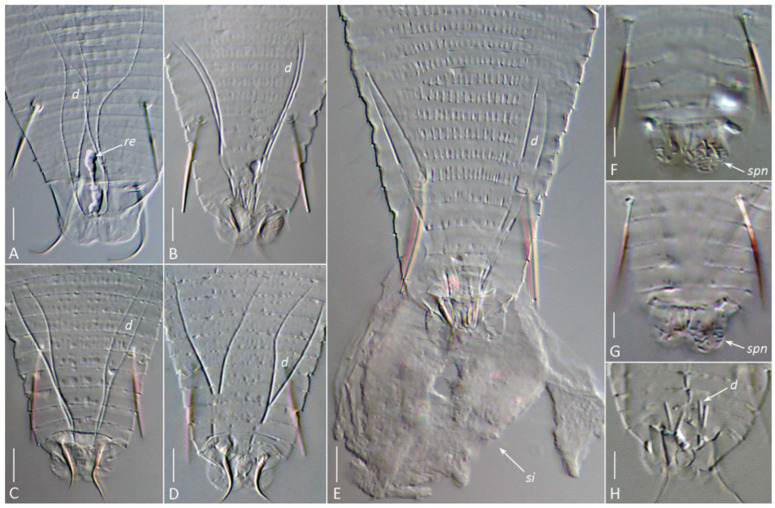
DIC images of the silk-producing apparatus in completely cleared (Hoyer’s medium) females (**A**–**G**) and males (**H**) of *Aceria aegyptindicae* (**A**) and *Aculops knorri* (**B**–**H**). Abbreviations: *d*—ducts of the anal glands; *re*—putative rectum filled with silk; *si*—silk extruded from the silk-producing organs of a slightly compressed mite; *spn*—areas of porose cuticle (the spinneret) on the caudal lobes. Scale bar: (**A**–**D**) 5 µm; (**E**–**H**) 3 µm.

In females of both species, a uniformly organized SPA was observed beneath the cuticle of the posterior opisthosoma (Figure 4E,F; Figure 6 and Figure 7). It comprised the same structures as those recently described in detail for the southern African web-spinning species *Ab. schotiae* [27], including large silk-producing anal glands extending along approximately one-fourth of the body length.

The visibility of specific SPA elements depended on the mounting method. In glycerin mounts of live *A. knorri* females, the elongated silk-producing anal glands and, rarely, the reservoirs for silk storage (cuticular sacs and rectum) were visible (Figure 6A–C). In partially cleared specimens (Hoyer’s medium), the outlines of the rapidly dissolving anal glands and traces of their cuticular ducts were observed (Figure 6D). 

Slight mechanical pressure applied to the cover slip with a dissection needle induced living *A. knorri* females in the early clearing process to extrude a droplet of silk material through the anus, forming a round silk deposit with a small central clearing at the anal opening (Figure 7E). In fully cleared specimens, a pair of two-branched anal gland ducts, remnants of the cuticular reservoirs between them (sometimes containing undissolved silk), and areas of porose cuticle on the anal lobes (the spinneret) were visible (Figure 7).

In males, the SPA was organized similarly to that of females but was generally smaller, with notably shorter anal gland ducts (Figure 7H).

### 3.3. Molecular Phylogenetics

#### 3.3.1. COI and 28S Sequence Diversity

All DNA isolates of *A. knorri* (covering two morphotypes observed in the population 1, see Section 3.2.1) yielded identical (K2P = 0) COI and 28S sequences, confirming the conspecificity of the analyzed specimens. 

COI sequences of *Cisaberoptus* and *Aceria* showed significant diversity (Table S4). Within the genus *Aceria*, three distinct clusters were identified: (i) isolates d392 and d398 of *Ac. aegyptindicae* from Vietnam (K2P = 0); (ii) isolate d395 of *Ac.* cf. *aegyptindicae* from Vietnam; and (iii) isolate PC22b of *Aceria* sp. from South Africa. The high genetic distance between two last clusters (K2P = 0.18) suggests that they may be cryptic species, each distinct from the Vietnamese lineage of *Ac. aegyptindicae* (distances of 0.20 and 0.23, respectively).

COI sequences of *Cisaberoptus* from Vietnam (isolates d400 and d401) and Florida (isolate d211) formed a genetically uniform cluster (K2P = 0), representing a single widespread species. However, the specimen from India (MW491351) was markedly divergent from this cluster (K2P = 0.24–0.25), indicating that it is a putative separate species within the genus.

K2P distances for the 28S gene revealed distinct patterns of sequence divergence (Table S5). Within *Aceria*, specimens from Hanoi identified as *Ac. aegyptindicae* (isolates d392, d393 and d398) and *Ac.* cf. *aegyptindicae* (isolates d395 and d1000) formed a highly homogeneous group (K2P = 0–0.007). In contrast, an *Aceria* specimen from South Africa (isolate d1063) was consistently divergent from all other *Aceria* sequences (K2P = 0.055–0.057), indicating that it represents a distinctly separate lineage. All analyzed specimens of *C. kenyae* from Florida (isolates d1060, d1061, d1062), South Africa (d1065, PC22a), and Vietnam (d400, d401, d636) were genetically uniform (K2P = 0–0.002), confirming their conspecificity across a broad geographic range.

#### 3.3.2. COI and 28S Phylogenetic Analyses (Figure 8)

Maximum likelihood analyses of COI and 28S sequences produced different overall tree topologies but shared several congruent, well-supported clades. Both analyses confirm the basal divergence of Eriophyoidea into three major lineages, Phytoptidae s. str., Nalepellidae, and Eriophyidae s. str., with poorly resolved internal relationships. Both datasets support the monophyly of the subfamily Cecidophyinae, placing it as sister to a lineage containing the monophyletic genus *Cisaberoptus* and a clade comprising mango-associated *Aceria* spp. The ecological group of web-producing species is not monophyletic; its members are dispersed, with one lineage (the mango-associated *Cisaberoptus* and *Aceria*) sister to Cecidophyinae, and another (comprising *Aberoptus* and *A. knorri*) positioned within clades dominated by members of Anthocoptinae and Aceriini. 

The COI and 28S trees differ in the inferred sister taxa for *A. knorri*. The 28S topology suggests an ecologically based relationship, grouping *A. knorri* with *Aceria litchii* (both associated with Sapindaceae). In contrast, the COI topology—lacking data for *Ac. litchii*—supports a taxonomic relationship, placing *A. knorri* as sister to a clade of three *Aculus* species (all members of the tribe Anthocoptini).

**Figure 8:**
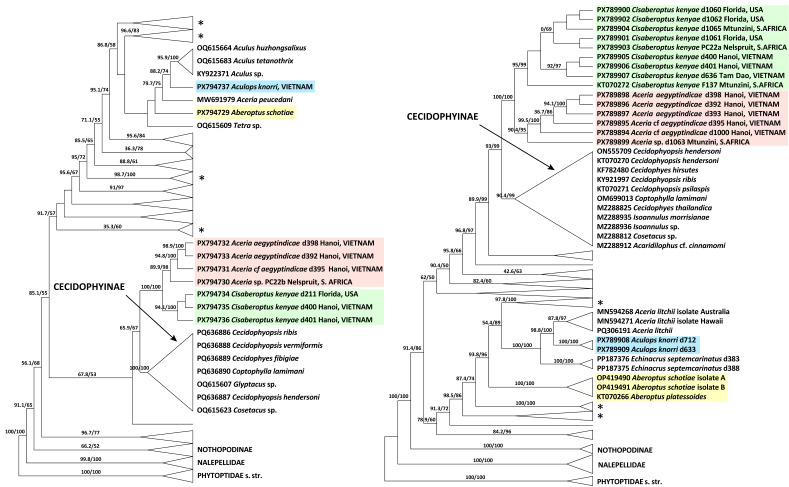
*COI* (**left**) and *28S* (**right**) ML phylogenies of Eriophyoidea showing the placement of *Aberoptus* (yellow), *Cisaberoptus* (green), *Aculops knorri* (blue), and mango-associated *Aceria* spp. (pink). Branches are labeled with aLRT values and UFBoot >50. Non-relevant clades are collapsed. Asterisks mark clades dominated by Anthocoptinae and Aceriini.

## 4. Discussion

This study, based on extensive material from Asia, Africa, and North America, revealed three central findings in web-spinning gall mites: (a) the convergent evolution of silk-webbing, (b) cryptic diversity within silk-producing lineages of Eriophyidae, and (c) cooperative web-production in sympatric eriophyid species associated with mango.

***Cisaberoptus*—*Aceria* sympatry on mango tree leaves.** Our data demonstrate that similar to web-spinning mites of genus *Aberoptus* [25,27], *Aceria aegyptindicae*, *Cisaberoptus kenyae* (co-existing on mango), and *Aculops knorri* (from *Lepisanthes rubiginosa*) share a suite of related ecological traits. They produce large protective silk films, feed and reproduce beneath them, establish their younger colonies under transparent webbing along leaf veins, later expanding the silk webbing into the interveinular spaces, and show colony density and web coverage increasing on older leaves (Figure 2 and Figure 3). A notable divergence, however, is evident in their phytopathological impact. Contrary to *A. knorri* and *Aberoptus*, which cause no visible damage to their hosts, the mixed *Cisaberoptus*–*Aceria* colonies on mango from Vietnam were consistently associated with necrotic epidermal patches beneath the webbing (Figure 3C)—a type of damage previously reported in India for *C. kenyae* [56]. These data indicate a more injurious interaction on mango resulting in leaf tissue damage, potentially due to mite saliva or transmitted infectious agents.

Reports from geographically distant populations indicate that *C. kenyae* and *Ac. aegyptindicae* often coexist in syntopy under a common web on mango leaves and exhibit synchronized seasonal population dynamics [34]. Furthermore, we found that *Ac. aegyptindicae* possesses a well-developed silk-producing apparatus (Figure 4E), suggesting a capability for building large protective web—a trait shared with *Cisaberoptus*, *Aberoptus*, and *A. knorri*. The frequent co-occurrence, population synchrony, web-spinning capability, and shared use of a web support the hypothesis of a symbiotic association of *C. kenyae* and *A. aegyptindicae* on mango. We consider this type of relationship a case of sporadic mutualism between *C. kenyae* and *A. aegyptindicae,* genetically very close sister species. When their populations develop in syntopy, these relationships appear to involve the cooperative production and maintenance of a common web, resembling the symbiotic relationships observed between populations of some other arachnids [57,58,59]. 

**Cryptic diversity in populations of web-spinning mites.** Morphological and molecular investigations revealed undocumented variation in populations of web-spinning eriophyids from *L. rubiginosa* and mango. For *A. knorri* in Vietnam, two morphotypes (“narrow” and “wider”) were distinguished by body proportions and ornamentation of the dorsal opisthosomal annuli, both diverging from the type population from Thailand, which is characterized by a smooth prodorsal shield. Interestingly, a very similar species, *Tetra lepisanthae* Boczek & Chandrapatya 2000 from Thailand, most probably synonymous with *A. knorri*, has two faint admedian lines on the prodorsal shield [60], like our specimens from Vietnam, suggesting the presence of a common population of *A. knorri* in Indochina. We also found subtle but consistent morphometric differences in *C. kenyae* and *Ac. aegyptindicae* from our material compared to other geographical populations. Furthermore, two putative forms were observed within the Vietnamese *Ac. aegyptindicae* specimens, slightly differing in prodorsal shield and genital coverflap ornamentation. 

Molecular data provided a crucial framework for interpreting this variation. For *A. knorri*, the genetic uniformity (K2P = 0) of COI and 28S sequences strongly indicates that the two morphotypes represent intraspecific variation, likely attributable to seasonal female dimorphism (deuterogeny) [61]. Conversely, for the mango-associated mites, molecular data clarified complex species boundaries. All *Cisaberoptus* specimens from Vietnam, Florida, and South Africa formed a single genetically uniform cluster, which suggest that *C. kenyae* may have been co-introduced globally with its host, *M. indica* (Figure 1), coinciding with the most intensive areas of contemporary mango cultivation across continents [43]. The divergent sequence (MW491351) of *Cisaberoptus* from India suggests a cryptic species within the genus, which may reflect genetic diversity and the presence of quasi-isolated old populations of mango in India—the center of origin for most contemporary mango cultivars [43,62,63].

For web-spinning *Aceria* associated with mango, the genetic data presented a more complicated picture: while Vietnamese *Ac. aegyptindicae* and *Ac.* cf. *aegyptindicae* were homogeneous in 28S, COI sequence analyses suggested that *Ac.* cf. *aegyptindicae* from Vietnam and an *Aceria* sp. from South Africa might be distinct cryptic species. This discordance between the mitochondrial and nuclear markers, as well as between morphology and COI data, highlights the potential for cryptic speciation and warrants further study with additional marker genes and populations.

**Convergent evolution of silk-webbing in Eriophyoidea.** The molecular phylogenetic analyses yielded two key insights into the evolution of web-spinning. First, the web- producing habit is a homoplasy; it has evolved at least twice independently within Eriophyidae—once in the lineage leading to *Aberoptus* and *A. knorri*, and once in the monophyletic, mango-associated *Cisaberoptus + Aceria* (mCA) lineage. This supports the hypothesis that web-spinning is a convergent adaptation, which evolved based on a symplesiomorphic internal anatomy [30] and resulted in the formation of a rectum-associated silk-producing apparatus (SPA) [27]. Our novel documentation of the SPA in *A. knorri* and *Ac. aegyptindicae* confirms its structural homology with that of *Aberoptus*, underscoring this convergence in phylogenetically remote clades.

Secondly, the consistent sister-group relationship between the web-spinning mCA lineage and the subfamily Cecidophyinae is intriguing, as it coincides with notable structural similarities in their external and internal genitalia. If confirmed, this relationship would provide a strong phylogenetic rationale for including the mCA clade within Cecidophyinae and for excluding the mango-associated *Aceria* species from the polyphyletic genus *Aceria*. The supported sister relationship between *C. kenyae* and *Ac. aegyptindicae* further suggests that they likely share a homologous SPA and web-spinning mechanism, though this will require direct morphological confirmation.

## 5. Conclusions

Our molecular and morphological evidence establishes the convergent evolution of silk-webbing in Eriophyoidea and reveals a novel, testable phylogenetic hypothesis linking web-spinning eriophyid mites from mango to Cecidophyinae. The following future perspectives will be crucial to testing the phylogenetic relationships proposed here and to build a comprehensive understanding of silk-use and evolution across Eriophyoidea: (a) a comparative histomorphology of *Cisaberoptus* and other web-spinning taxa, (b) phylogenetics based on new molecular datasets, including more conserved sequences such as single-copy nuclear protein genes, (c) investigation of silk genetics and proteomics, (d) targeted searches for new web-spinning taxa, and (e) experimental studies elucidating the functions of silk in eriophyoids that do not produce large web nests.

## Figures and Tables

**Table 1:** Material from *Lepisanthes rubiginosa* and *Mangifera indica* used for DNA extraction (N—number of mites used for extracting DNA).

Mite Species	DNA Isolate	N	28S	COI	Collecting Details and Designation in Figure 1 (In Brackets)
*Aculops* *knorri*	d712	5	PX789908	PX794737	VIETNAM: Hoa Xuan Commune, Dak Lak Province, 12°52′43.8″ N 109°25′20.7″ E, 5 March 2024, coll. V.T. Nguyen, (9)
	d633	5	PX789909	PX794738	
	d1057	1	PX789910	PX794739	
	d1059	1	PX789911	PX794740	
*Cisaberoptus* *kenyae*	d400	3	PX789905	PX794735	VIETNAM: Hanoi, 21°04′18.8″ N, 105°46′30.2″ E, 14 February 2023, coll. P.E. Chetverikov, (8)
	d401	3	PX789906	PX794736	
	d636	5	PX789907	―	VIETNAM: Tam Dao, 21°27′59.9″ N, 105°34′51.5″ E, 11 March 2024, coll. P.E. Chetverikov, (8)
	d1060	1	PX789900	―	USA: Florida, Daytona Beach, 29°10′17.0″ N, 80°58′54.1″ W, 8 February 2019, coll. M. Cain, (4)
	d1061	4	PX789901	―	
	d1062	3	PX789902	―	
	d211	8	―	PX794734	
	PC22a	1	PX789903	―	SOUTH AFRICA: Nelspruit, 25°27′08.2″ S, 30°58′11.5″ E, 17 March 2017, coll. C. Craemer and S. Neser, (6)
	d1065	2	PX789904	―	SOUTH AFRICA: Mtunzini, 28°57′24.4″ S, 31°44′54.6″ E, 10 March 2013, coll. C. Craemer and S. Neser, (5)
*Aceria* *aegyptindicae*	d392	1	PX789896	PX794733	VIETNAM: Hanoi, 21°04′18.8″ N, 105°46′30.2″ E, 14 February 2023, coll. P.E. Chetverikov, (8)
	d393	1	PX789897	―	
	d398	1	PX789898	PX794732	
*Aceria* cf. *aegyptindicae*	d1000	2	PX789894	―	
	d395	2	PX789895	PX794731	
*Aceria* sp.	PC22b	1	―	PX794730	SOUTH AFRICA: Nelspruit, 25°27′08.2″ S 30°58′11.5″ E, 17 March 2017, coll. C. Craemer and S. Neser, (6)
	d1063	1	PX789899	―	SOUTH AFRICA, Mtunzini, 28°57′24.4″ S, 31°44′54.6″ E, 10 March 2013, coll. C. Craemer and S. Neser, (5)

## Data Availability

The new sequences of eriophyoid mites have been deposited in the National Center for Biotechnology Information (NCBI) GenBank database (https://www.ncbi.nlm.nih.gov/genbank (accessed on 10 January 2026); accession numbers: PX789894-PX789911, PX794729-PX794740.

## Supplementary Materials

The following supporting information can be downloaded at: https://www.mdpi.com/article/10.3390/insects17030259/s1, Figure S1: Tibial seta *l*’ I in two females of *Cisaberoptus kenyae*; Table S1: Morphological differences between females of *A. knorri* from Thailand [38] and Vietnam; Table S2: Morphological differences between females of *C. kenyae* from Kenya [45], Egypt [34], Vietnam, and Florida; Table S3: Morphological differences between females of *Ac. aegyptindicae* from Egypt [34], Brazil [37], and Vietnam; Table S4: K2P genetic distances for the COI gene sequences among studied isolates of *Aceria* and *Cisaberoptus* from mango; Table S5: K2P genetic distances for the 28S gene sequences among the studied isolates of *Aceria* and *Cisaberoptus* from mango.
