# Supplementary material for: Convergent Evolution of Silk Webbing in Eriophyoid Mites (Eriophyoidea) and Aceria–Cisaberoptus Sympatry on Mango
Source: Insects. 2026 Feb 28;17(3):259. doi: 10.3390/insects17030259 (PMC13027148; doi:10.3390/insects17030259)
Supplement: Supplementary file 1 [file insects-17-00259-s001.zip › Supplementary Tables 1_2_3_4_5___REVISED_for_PROOF.pdf]

Table S1. Morphological differences between females of *A. knorri* from Thailand and Vietnam.

Characters	Specimens from Thailand (n – ?)	Specimens from Vietnam	
		"Narrower" females (n=5)	"Wider" females (n=5)
Ornamentation of prodorsal shield	No lines	Two admedian lines	Two admedian lines
Microtubercles on dorsal annuli	Absent	Absent (Fig. 4H)	Present (Fig. 4G)
Length of body	128–148	174 (166–188)	176 (170–180)
Width of body	45–50	56 (52–58)	63 (60–65)
Width/length ratio	no data	0,32 (0,30–0,34)	0,36 (0,35–0,36)
Length of *l'* I	7.5	5 (4–6)	4 (3–5)
Length of *l''* I	no data	20 (17–21)	23 (22–24)
Length of *2a*	no data	25 (22–27)	30 (27–31)
Number of ventral annuli	53	60 (58–64)	57 (54–59)
Length of *c2*	24	31 (29–33)	32 (31–33)
Length of *d*	22	32 (30–34)	31 (29–34)
Length of *h2*	17	10 (9–10)	9 (8–10)
Reference	[38]	this study	this study

Table S2. Morphological differences between females of *C. kenyae* from Kenya, Egypt, Vietnam and Florida.

Characters	Specimens from Kenya (n – ?)	Specimens from Egypt (n=15)	Specimens from Vietnam (n=5)	Specimens from Florida (n=3)
Length of body	190–210	195–212	197 (183–215)	175 (171–180)
Width of body	52	54–58	71 (70–72)	63 (60–65)
Length of prodorsal shield	32 [1]	30–33 [1]	35 (32–39)	32 (30–35)
Width of prodorsal shield	38 [1]	35–45 [1]	63 (61–65)	53 (48–57)
Length of *sc*	17	15–18	22 (19–26)	15 (12–17)
Number of *em* II rays	17	17	15 (15–16)	15 (14–15)
Length of *h2*	no data	30–37	51 (48–52)	30 (24–41)
Reference	[45]	[34]	this study	this study

[1] A comparison with the original description of *C. kenyae*, reporting a body width of 52 µm [45], and the width-to-length proportions in fig. 1 DA, suggests an error in the prodorsal shield measurements given by Keifer [45]. This same reasoning likely applies to the prodorsal shield measurements reported by Elhalawany et al. [34].

Table S3. Morphological differences between females of *Ac. aegyptindicae* from Egypt, Brazil and Vietnam.

Characters	Specimens from Egypt (n=10)	Specimens from Brazil (n=5)	Specimens from Vietnam (n=5)
Width of prodorsal shield	32–44	38–44	34–36
Length of *sc*	14–20	16–19	16–18
Distance between *sc*	25–28	20–22	25–28
Length of *ft''* I	10–12	14–17	14–16
Length of *l''* I	14–17	18–22	21–22
Length of *ft'* II	7–9	4–5	5–7
Length of *ft''* II	10–12	15–18	15–17
Length of *l''* II	8–10	5–7	7–8
Length of *1a*	7–8	11–14	12–14

Length of *2a*	17–19	35–39	30–32
Distance between *2a*	23–25	22–23	21–24
Length of genital coverflap	14–15	10–11	11–13
Width of genital coverflap	20–21	23–24	22–24
Number of ventral annuli	56–62	45–50	51–56
Length of *c2*	11–13	10–15	15–21
Length of *d*	28–35	52–59	50–57
Length of *e*	24–32	43–50	37–40
Length of *h2*	25–35	36–40	53–74
Reference	[34]	[37]	this study

Table S4. K2P genetic distances for the COI gene sequences among studied isolates of *Aceria* and *Cisaberoptus* from mango. Isolate codes are given in Table 1.

#	Sequence	1	2	3	4	5	6	7	8
1	PX794732 *A. aegyptindicae* d398 Hanoi	—							
2	PX794733 *A. aegyptindicae* d392 Hanoi	0	—						
3	PX794731 *A.* cf *aegyptindicae* d395 Hanoi	0,20	0,20	—					
4	PX794730 *Aceria* sp. PC22b S.Africa	0,23	0,23	0,18	—				
5	PX794735 *C. kenyae* d400 Hanoi	0,27	0,27	0,25	0,24	—			
6	PX794736 *C. kenyae* d401 Hanoi	0,27	0,27	0,25	0,24	0	—		
7	PX794734 *C. kenyae* d211 Florida	0,27	0,27	0,26	0,25	0	0	—	
8	MW491351 *C. kenyae* India	0,24	0,24	0,21	0,21	0,24	0,24	0,25	—

Table S5. K2P genetic distances for the 28S gene sequences among studied isolates of *Aceria* and *Cisaberoptus* from mango. Isolate codes are given in Table 1.

#	Sequence	1	2	3	4	5	6	7	8	9	10	11	12	13	14	15
1	PX789896 *A. aegyptindicae* d392 Hanoi	—														
2	PX789897 *A. aegyptindicae* d393 Hanoi	0	—													
3	PX789898 *A. aegyptindicae* d398 Hanoi	0	0	—												
4	PX789895 *A.* cf *aegyptindicae* d395 Hanoi	0,003	0,003	0,003	—											
5	PX789894 *A.* cf *aegyptindicae* d1000 Hanoi	0,007	0,007	0,007	0,004	—										
6	PX789899 *Aceria* sp. d1063 Mtzini S. Africa	0,055	0,055	0,055	0,056	0,057	—									
7	PX789900 *C. kenyae* d1060 Florida	0,063	0,063	0,063	0,064	0,065	0,070	—								
8	PX789901 *C. kenyae* d1061 Florida	0,063	0,063	0,063	0,064	0,065	0,070	0	—							
9	PX789902 *C. kenyae* d1062 Florida	0,063	0,063	0,063	0,064	0,065	0,070	0	0	—						
10	PX789903 *C. kenyae* PC22a Nelspruit S. Africa	0,063	0,063	0,063	0,064	0,065	0,070	0	0	0	—					
11	PX789904 *C. kenyae* d1065 Mtzini S. Africa	0,063	0,063	0,063	0,064	0,065	0,070	0	0	0	0	—				
12	KT070272 *C. kenyae* F137 Mtzini S. Africa	0,064	0,064	0,064	0,065	0,066	0,071	0,001	0,001	0,001	0,001	0,001	—			
13	PX789905 *C. kenyae* d400 Hanoi	0,064	0,064	0,064	0,065	0,066	0,071	0,001	0,001	0,001	0,001	0,001	0,002	—		
14	PX789906 *C. kenyae* d401 Hanoi	0,064	0,064	0,064	0,065	0,066	0,071	0,001	0,001	0,001	0,001	0,001	0,002	0	—	
15	PX789907 *C. kenyae* d636 Tam Dao	0,064	0,064	0,064	0,065	0,066	0,071	0,001	0,001	0,001	0,001	0,001	0,002	0	0	—
