# Supplementary figures and images for: Convergent Evolution of Silk Webbing in Eriophyoid Mites (Eriophyoidea) and Aceria–Cisaberoptus Sympatry on Mango
Source: Insects. 2026 Feb 28;17(3):259. doi: 10.3390/insects17030259 (PMC13027148; doi:10.3390/insects17030259)

**Fig. S1.** Tibial seta I' I in two females of *Cisaberoptus kenya* (DIC LM)

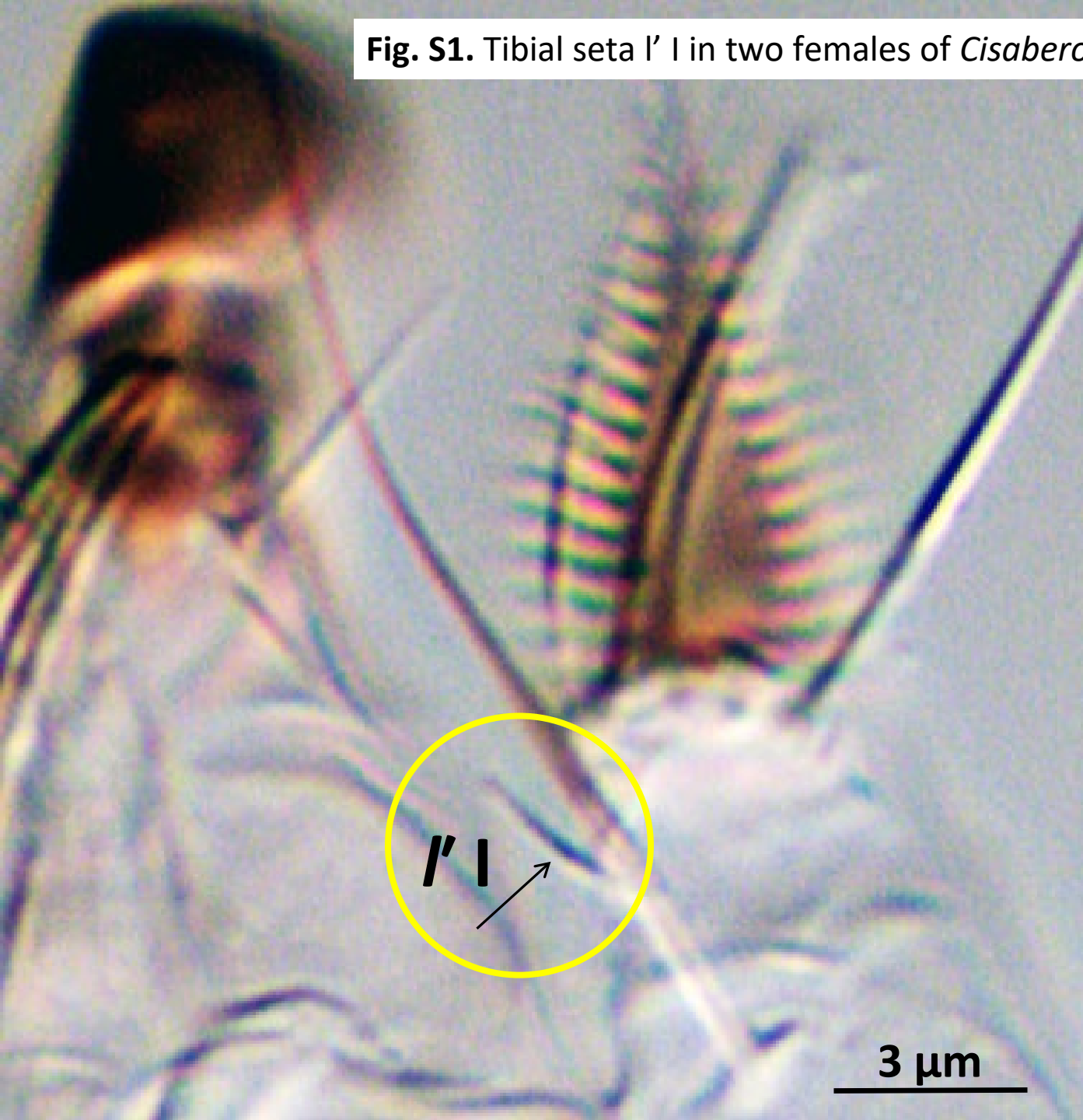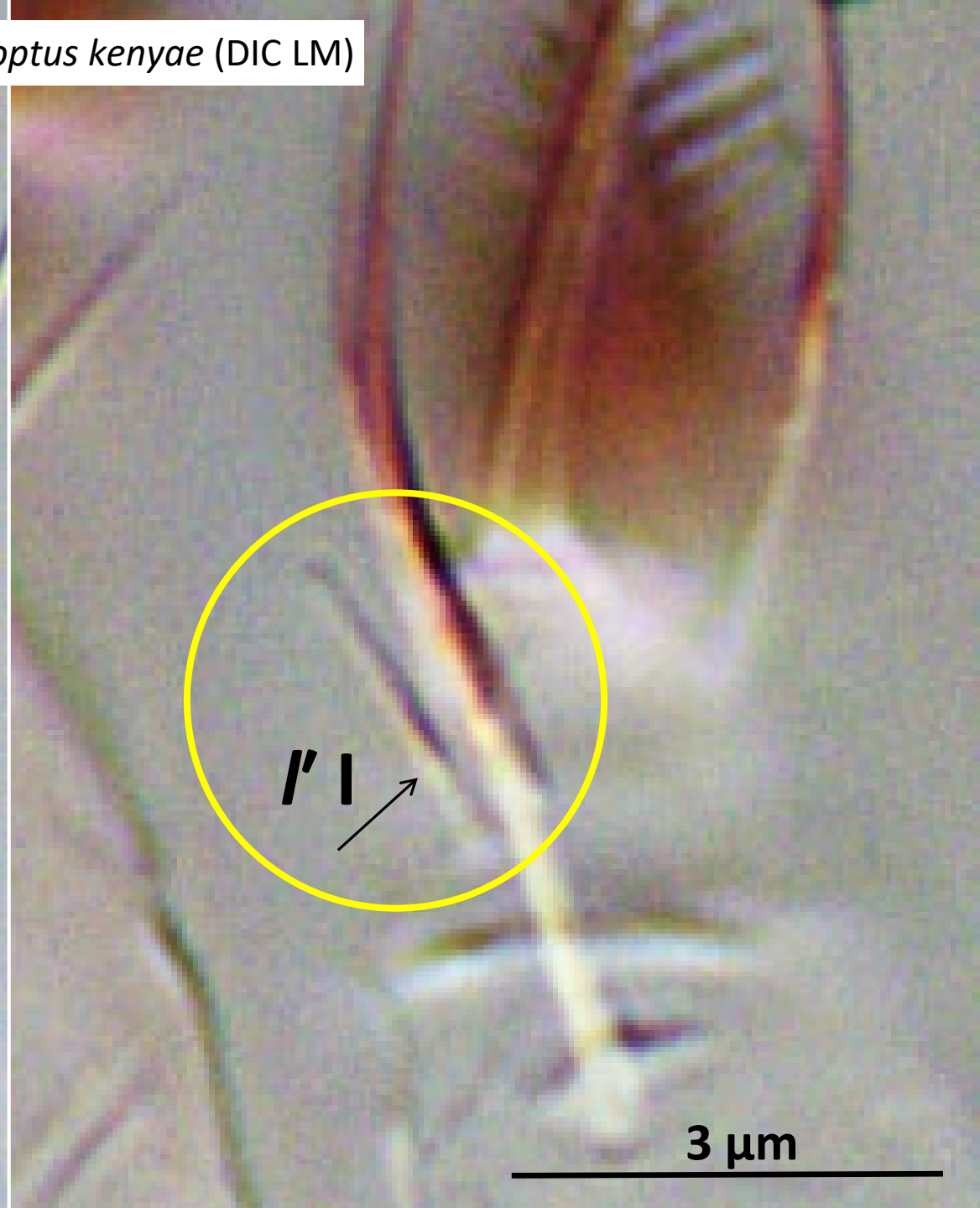

Supplement: Supplementary file 1 [file insects-17-00259-s001.zip › SFig1_s_tib_1.pdf]
